# Coiled Internal Carotid Arteries Associated with Bilateral Sequential Strokes

**DOI:** 10.1155/2013/929530

**Published:** 2013-05-16

**Authors:** Gary G. Tse, Elna M. Masuda, Aaron M. McMurtray, Beau K. Nakamoto

**Affiliations:** ^1^Department of Medicine, University of Hawaii, 1356 Lusitana Street, 7th Floor, Honolulu, HI 96813, USA; ^2^Straub Clinics and Hospital, 888 South King Street, Honolulu, HI 96813, USA; ^3^Department of Surgery, University of Hawaii, 1356 Lusitana Street, 6th Floor, Honolulu, HI 96813, USA; ^4^Department of Neurology, Los Angeles Biomedical Research Institute at Harbor-UCLA, Harbor-UCLA Medical Center, 1000 W. Carson Street, Building N25, Torrance, CA 90509, USA

## Abstract

The risk of stroke and management of coiling of the cervical internal carotid artery in the absence of an atherosclerotic carotid bulb lesion is unclear. We report a case of an otherwise healthy 39-year-old woman who developed bilateral sequential strokes associated with bilateral coiled internal carotid arteries. We discuss the risk of stroke and management of coiled carotid arteries as they relate to the patient presented.

## 1. Introduction

Elongation of the internal carotid artery (ICA) can result in tortuosity, coiling, and kinking. The associated stroke risk and management of these vascular anomalies is unclear. We report a case of a 39-year-old female who presented with bilateral sequential strokes associated with 360-degree coil in both cervical ICAs.

## 2. Case Presentation

**Figure 1:**
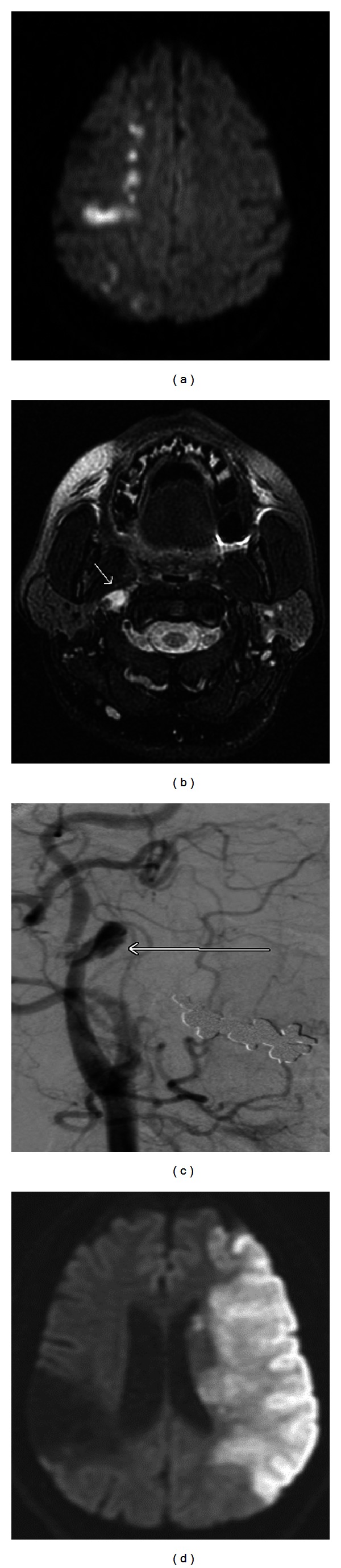
((a)–(d)) 39-year-old female presenting with acute onset of a right-sided headache associated with left hemiparesthesia and hemiparesis following sexual intercourse. (a) Initial brain MRI axial DWI sequence demonstrates abnormal restricted diffusion in a watershed distribution of the right cerebral hemisphere consistent with an acute stroke. (b) Axial T1-weighted fat-saturated sequence of the neck vessels demonstrates a hyperintense signal abnormality within the subintimal space of the right internal carotid artery (arrow) consistent with a dissection. (c) Conventional angiogram of the right internal carotid artery demonstrates a 360° loop with an associated fusiform aneurysm filled with thrombus (arrow). (d) Patient presented with acute onset of right hemiplegia and global aphasia 8 years after her first stroke. Brain MRI axial DWI sequence demonstrates a large region of abnormal restricted diffusion in the left cerebral hemisphere consistent with an acute stroke in the left middle cerebral artery distribution. There is encephalomalacia of the right parietal lobe in the region of her prior stroke.

A 39-year-old female presented to the emergency department with acute onset of right retroorbital and temporal headache associated with left-sided weakness and numbness following sexual intercourse. She was otherwise healthy without cerebrovascular risk factors, tobacco use, or illicit drug use. She was taking oral contraceptives. There was no family history of premature coronary artery disease or stroke. Initial general examination was notable for a blood pressure of 158/98. Neurologic examination revealed a left lower facial droop and hemiparesis. Brain MRI revealed an acute stroke in the right cerebral hemisphere in a watershed distribution (Figure 1(a)). Head MRA demonstrated no flow-related enhancement within the petrous and cavernous segments of the right ICA with reconstitution of the ICA at the ophthalmic artery level. Axial fat-saturated T1-weighted MRI through the skull base demonstrated a distortion of the lumen of the petrous and cavernous segments of the right ICA by a rounded region of increased signal abnormality consistent with a dissection (Figure 1(b)). Conventional 4-vessel cerebral angiography demonstrated 360-degree loops in both cervical ICAs (Figure 1(c)). At the apex of the loop involving the proximal right internal carotid artery, there was a fusiform aneurysm filled with a subintimal thrombus resulting in poor antegrade flow into the distal ICA and cerebral circulation. The 360-degree loop in the mid-cervical portion of the left internal carotid artery was not associated with outflow obstruction. The 360-degree loop in the distal right ICA was not felt to be amenable to surgical intervention by vascular surgery due to the distal nature of the vascular anomaly. She was anticoagulated with warfarin for 7 months followed by treatment with extended-release dipyridamole plus aspirin daily. Her left hemiparesis progressively improved following discharge and she had no residual weakness after 1 month. Eight years later, she presented with acute onset of right hemiplegia associated with a global aphasia. Brain MRI revealed a large stroke in the distribution of the left middle cerebral artery (Figure 1(d)). She was discharged four months later without clinical improvement and was lost to followup.

## 3. Discussion

Elongation of the ICA is a relatively common vascular anomaly that occurs during embryological development. The ICA originates from the third aortic arch and dorsal aorta during embryological development. If the ICA does not straighten as the heart and large vessels migrate into the developing thorax during embryological development, it remains elongated in its angulated state and becomes tortuous, coiled, or kinked [1]. In one of the largest angiographic series of 2453 carotid angiograms, tortuosity of the ICA was observed in 489 (35%), coiling in 88 (6%), and kinking in 65 (5%) [2]. A tortuous ICA is an elongated ICA with an exaggerated C- or S-shaped curving. Coiling is defined as ICA elongation causing a circular configuration. Kinking is a variant of coiling and describes an elongated cervical ICA segment with an angulation less than 90°. Kinked ICA can be further classified based on the severity of angulation: Grade 1 (mild) is angled at 90°–60°, Grade 2 (moderate) at 60°–30°, and Grade 3 (severe) less than 30° [2, 3].

The natural history of elongated ICAs and the associated stroke risk is unclear. In the older population, the frequent presence of concomitant atherosclerotic disease at the carotid bifurcation confounds a causal relationship between these vascular anomalies and stroke. In individuals with elongated ICAs without concomitant atherosclerosis at the carotid bifurcation, it is generally assumed that ICA tortuosity should be considered a relatively benign condition while kinking of the ICA may be associated with cerebrovascular symptoms in the anterior circulation of the cerebral hemispheres. This hypothesis is supported by experimental studies demonstrating that blood flow may be reduced by >40% with an angle of 60° and by >60% with an angle of 30° [4]. There is a lack of consensus as to how to identify which individuals should be considered for surgical intervention and which surgical procedure to perform. In general, however, individuals with a kinked ICA without atherosclerosis causing an ICA stenosis of 70% or greater by North America Symptomatic Carotid Endarterectomy Trial criteria or asymptomatic subjects with a kink angle less than 30% and occlusion of the contralateral carotid artery could be considered for surgical intervention [5]. Common approaches include ICA transection at the origin and reimplantation at the carotid bulb, resection of the ICA and transposition onto the external carotid artery, and ICA resection with insertion of a common-to-internal carotid saphenous or polytetrafluoroethylene graft [6]. Studies comparing stroke risk in individuals surgically treated with transection of the kinked ICA at the origin and reimplantation at the carotid bulb versus medical therapy with aspirin are mixed, which may be due in part to differences in study design and population [7, 8].

Little is known about the significance, associated stroke risk, and suggested management of coiled ICAs. Our subject with bilateral 360-degree coiled ICAs initially presented with an ICA dissection followed 8 years later by a stroke to the contralateral cerebral hemisphere. Histologic studies of coiled and kinked ICAs have revealed either fibromuscular dysplasia, which consists of thickened fibromuscular hyperplasia alternating with areas of pronounced thinning of the media with superimposed intimal fibromuscular hyperplasia, or metaplasia, which consists of significantly reduced tunica intima and media layers with a compensatory increase of well-differentiated loose connective tissue resulting in increased laxity of the extracranial ICA wall [7, 8]. These histologic findings are intriguing given that both fibromuscular hyperplasia or metaplasia may have predisposed our patient to the development of a dissection secondary to weakening of the ICA endothelial layer. Indeed, La Babera et al. found intramural hemorrhage on macroscopic examination of two out of ten ICAs from individuals who underwent surgical intervention of an elongated ICA [9]. Furthermore, the endothelial alterations seen in histological studies of coiled and kinked ICAs may have been a source of thromboembolism and the cause for our patient's second stroke 8 years later. 

Management of a possibly symptomatic coiled ICA without concomitant atherosclerosis has been given little attention unless there is an associated significant stenosis by carotid duplex ultrasound. Our case did not have an associated stenosis with the coiled ICA and was therefore managed with anticoagulation with warfarin for 7 months followed by extended-release dipyridamole plus aspirin for her dissection, according to the 2011 American Stroke Association Guidelines [10]. Despite this, she went on to experience a presumed thromboembolic stroke to the contralateral hemisphere 8 years later. Again, the subject was not felt to be a candidate for surgical intervention given the distal location of the 360-degree coil.

In summary, we describe a case of an otherwise healthy, young female with bilateral 360-degree coiled ICAs who presented with a carotid dissection followed by a presumed thromboembolic stroke to the contralateral hemisphere 8 years later. While no treatment recommendations can be suggested from this single report, our case highlights that the presence of a coiled ICA should be considered in the differential diagnosis of stroke in a younger individual.

## Abbreviations

ICA: Internal carotid artery.
